# Effects of diabetes definition on global surveillance of diabetes prevalence and diagnosis: a pooled analysis of 96 population-based studies with 331 288 participants

**DOI:** 10.1016/S2213-8587(15)00129-1

**Published:** 2015-08

**Authors:** NCD Risk Factor Collaboration (NCD-RisC)

## Abstract

**Background:**

Diabetes has been defined on the basis of different biomarkers, including fasting plasma glucose (FPG), 2-h plasma glucose in an oral glucose tolerance test (2hOGTT), and HbA_1c_. We assessed the effect of different diagnostic definitions on both the population prevalence of diabetes and the classification of previously undiagnosed individuals as having diabetes versus not having diabetes in a pooled analysis of data from population-based health examination surveys in different regions.

**Methods:**

We used data from 96 population-based health examination surveys that had measured at least two of the biomarkers used for defining diabetes. Diabetes was defined using HbA_1c_ (HbA_1c_ ≥6·5% or history of diabetes diagnosis or using insulin or oral hypoglycaemic drugs) compared with either FPG only or FPG-or-2hOGTT definitions (FPG ≥7·0 mmol/L or 2hOGTT ≥11·1 mmol/L or history of diabetes or using insulin or oral hypoglycaemic drugs). We calculated diabetes prevalence, taking into account complex survey design and survey sample weights. We compared the prevalences of diabetes using different definitions graphically and by regression analyses. We calculated sensitivity and specificity of diabetes diagnosis based on HbA_1c_ compared with diagnosis based on glucose among previously undiagnosed individuals (ie, excluding those with history of diabetes or using insulin or oral hypoglycaemic drugs). We calculated sensitivity and specificity in each survey, and then pooled results using a random-effects model. We assessed the sources of heterogeneity of sensitivity by meta-regressions for study characteristics selected a priori.

**Findings:**

Population prevalence of diabetes based on FPG-or-2hOGTT was correlated with prevalence based on FPG alone (r=0·98), but was higher by 2–6 percentage points at different prevalence levels. Prevalence based on HbA_1c_ was lower than prevalence based on FPG in 42·8% of age–sex–survey groups and higher in another 41·6%; in the other 15·6%, the two definitions provided similar prevalence estimates. The variation across studies in the relation between glucose-based and HbA_1c_-based prevalences was partly related to participants' age, followed by natural logarithm of per person gross domestic product, the year of survey, mean BMI, and whether the survey population was national, subnational, or from specific communities. Diabetes defined as HbA_1c_ 6·5% or more had a pooled sensitivity of 52·8% (95% CI 51·3–54·3%) and a pooled specificity of 99·74% (99·71–99·78%) compared with FPG 7·0 mmol/L or more for diagnosing previously undiagnosed participants; sensitivity compared with diabetes defined based on FPG-or-2hOGTT was 30·5% (28·7–32·3%). None of the preselected study-level characteristics explained the heterogeneity in the sensitivity of HbA_1c_ versus FPG.

**Interpretation:**

Different biomarkers and definitions for diabetes can provide different estimates of population prevalence of diabetes, and differentially identify people without previous diagnosis as having diabetes. Using an HbA_1c_-based definition alone in health surveys will not identify a substantial proportion of previously undiagnosed people who would be considered as having diabetes using a glucose-based test.

**Funding:**

Wellcome Trust, US National Institutes of Health.

## Introduction

Diabetes prevalence and diabetes-related deaths are rising in most parts of the world, at least partly fuelled by the worldwide increase in excess weight and adiposity.^1^, ^2^, ^3^, ^4^, ^5^ This trend has created concerns about the health and functional consequences for patients, and costs for health systems.^6^, ^7^, ^8^ Tracking the epidemic and the progress of programmes aimed at reducing diabetes and its complications requires consistent and comparable measurement of the prevalence of diabetes and the coverage of drug and lifestyle interventions that slow diabetes progression and decrease the risk of complications.

Different biomarkers have been used to define diabetes, including fasting plasma glucose (FPG), 2-h plasma glucose in an oral glucose tolerance test (2hOGTT), and, more recently, HbA_1c_.^9^, ^10^, ^11^, ^12^, ^13^, ^14^, ^15^ Population-based health surveys in different countries and at different times have also used different biomarkers for glycaemia and diabetes, and thus define diabetes differently. The variety of biomarkers and definitions creates a challenge in consistently analysing diabetes prevalence across countries and over time, and in measuring what proportion of people with diabetes are diagnosed and receive effective treatments for diabetes and its complications.^1^, ^16^, ^17^ Therefore, there is a need to understand how the use of different biomarkers and definitions affects the identification of diabetes cases and the resulting estimates of population prevalence. This need is particularly pressing because two of the nine global targets for non-communicable diseases set after the 2011 United Nations high-level meeting on non-communicable diseases require estimates of diabetes prevalence: to halt the rise in the prevalence of diabetes, and to achieve a 50% coverage of drug treatment and counselling, including glycaemic control, to prevent coronary heart disease and stroke in people at high risk of cardiovascular disease.^4^, ^18^ Diabetes is also one of the four main non-communicable diseases for which there is a global target of 25% reduction in premature mortality by 2025 compared with 2010.^4^, ^18^

**Research in context**

**Evidence before this study**

We reviewed studies included in the NCD Risk Factor Collaboration databases for comparisons of various diabetes definitions. We also searched PubMed with the term ((A1c[Title/Abstract]) AND Sensitivity[Title/Abstract]) AND Specificity[Title/Abstract]) on April 13, 2015. We also searched the references of recent reviews and guidelines. We found some studies on the classification of individuals as having diabetes or on comparison of prevalence estimates based on different definitions in specific cohorts, especially for HbA_1c_ compared with either fasting plasma glucose (FPG) or 2-h oral glucose tolerance test (2hOGTT). Most of these analyses were based on a single cohort and very few covered different world regions. Two pooled analyses of Asian and European cohorts, and a study in the Pacific and Indian Ocean islands, assessed how the prevalence of diabetes and the classification of individuals as having diabetes versus not having diabetes changed depending whether diabetes was based on FPG or on 2hOGTT. There is no pooling study for HbA_1c_ and we identified only one review of data from six countries. Other studies compared different diabetes definitions among people with specific pre-existing diseases—eg, heart disease and tuberculosis. We also found some prospective studies that assessed how HbA_1c_ predicts future incidence of diabetes or cardiovascular diseases with mixed results.

**Added value of this study**

This study is the first pooling of a large number of population-based data from different world regions that addresses how different definitions of diabetes affect both the total prevalence, and the identification of previously undiagnosed individuals. By pooling a large number of data sources, the overall meta-analytical finding overcomes between-study variation, which can be probed in meta-regressions. Furthermore, by having a large number of studies, and age–sex groups within each study, we were able to develop regressions to convert across different diabetes definitions, which is essential for enhancing comparability over time and across countries in surveillance.

**Implications of all the available evidence**

The use of HbA_1c_ in surveillance requires further consideration in terms of how it predicts, and helps prevent, diabetes complications and sequelae. As such studies are done, and to maximise comparability of results across surveys, the best approach in population-based health surveys is to measure FPG and define diabetes as FPG 7·0 mmol/L or more or history of diagnosis with diabetes or using insulin or oral hypoglycaemic drugs, as used in the global monitoring framework for prevention and control of non-communicable diseases. When HbA_1c_ is used, it would be valuable to also measure FPG in a subsample of participants to provide information about how the two tests relate. The conversion regressions developed here can be used to convert prevalence based on FPG to that based on FPG-or-2hOGTT.

Some studies have analysed the classification of individuals as having diabetes or compared prevalence estimates based on different definitions in specific cohorts, especially for HbA_1c_ compared with either FPG or 2hOGTT.^19^, ^20^, ^21^, ^22^, ^23^, ^24^, ^25^, ^26^, ^27^, ^28^, ^29^, ^30^, ^31^, ^32^, ^33^, ^34^, ^35^, ^36^, ^37^, ^38^, ^39^, ^40^, ^41^, ^42^, ^43^, ^44^, ^45^, ^46^, ^47^, ^48^, ^49^, ^50^, ^51^, ^52^, ^53^, ^54^, ^55^, ^56^, ^57^, ^58^, ^59^, ^60^, ^61^ Most of these analyses were based on a single cohort and very few covered different geographical regions. Two pooled analyses of Asian and European cohorts, and a study in the Pacific and Indian Ocean islands, assessed how the prevalence of diabetes and the classification of individuals as having diabetes versus not having diabetes changed depending on whether diabetes was defined by FPG or 2hOGTT.^62^, ^63^, ^64^, ^65^, ^66^ There is no pooling study for HbA_1c_, which can be measured easily in population-based surveys without the need for overnight fasting and has been approved by the American Diabetes Association and WHO as a diagnostic test for diabetes.^11^, ^14^ However, a review of data from six countries reported that the sensitivity of diabetes diagnosis based on HbA_1c_ compared with FPG ranged from 17% to 78%,^67^ raising concerns about ethnic variation of HbA_1c_-based definition.^17^

We assessed the effect of diagnostic definitions both on the identification of diabetes in previously undiagnosed individuals and on the population prevalence estimates for diabetes in a pooled analysis of data from population-based health examination surveys in different world regions.

## Methods

### Study design

We aimed to answer two questions. First, how does the estimated prevalence of diabetes in a population change when the new definition of diabetes based on HbA_1c_ is used compared with earlier definitions based on blood glucose? Second, how does the new definition of diabetes based on HbA_1c_ compare with earlier definitions in identifying previously undiagnosed people with diabetes, as measured by the sensitivity and specificity of the new definition with respect to the previous ones? We further assessed whether sensitivity varied by the characteristics of the study population, because this possible variation is a source of concern about the generalisability of HbA_1c_ as a diagnostic and surveillance measure.^17^, ^67^, ^68^, ^69^, ^70^

For the HbA_1c_-based definition of diabetes, we used HbA_1c_ of 6·5% or more, or history of diagnosis with diabetes or using insulin or oral hypoglycaemic drugs.^11^ For definitions based on blood glucose, we used either the American Diabetes Association definition of FPG of 7·0 mmol/L or more, or history of diagnosis with diabetes or using insulin or oral hypoglycaemic drugs (which is also used in the global monitoring framework for prevention and control of non-communicable diseases),^12^, ^18^ or the WHO definition of FPG of 7·0 mmol/L or more, or 2hOGTT of 11·1 mmol/L or more, or history of diabetes or using insulin or oral hypoglycaemic drugs.^9^, ^10^

### Data sources

We used population-based data collated by the NCD Risk Factor Collaboration (NCD-RisC), a worldwide network of health researchers and practitioners who, together with WHO, have collated a large database of population-based health examination surveys and epidemiological studies of cardiometabolic risk factors. All data sources were checked by at least two independent reviewers as being representative of a national, subnational, or community population, and for study quality indicators such as fasting duration and the protocol for OGTT. We excluded surveys that had not used a standard glucose load for OGTT. Within each survey, we included participants aged 18 years and older who were not pregnant and had fasted at least for 6 h before measurement as a part of the survey instructions. We excluded HbA_1c_ data from before the year 2000 to minimise the use of non-standard assays.^71^ We also excluded surveys that had measured a biomarker only among participants with a high value of another—eg, studies in which FPG was only measured in participants with HbA_1c_ above a prespecified value, because the relation between the two measurements might be different in this prescreened group compared with the whole sample. The appendix shows details of individual surveys.

We restricted the analysis of sensitivity and specificity to people without a history of diabetes diagnosis, because previous diagnosis and the use of drug treatments probably affect the concentrations of biomarkers used to diagnose diabetes. History of diabetes diagnosis was established with survey-specific questions, such as “have you ever been told by a doctor or other health professional that you have diabetes?” or the combination of “do you now have, or have you ever had diabetes?” and “were you told by a doctor that you had diabetes?”. We also excluded follow-up surveys of closed cohorts from the analysis of sensitivity and specificity because active surveillance within a cohort shifts participants from undiagnosed to diagnosed status at each follow-up, thus affecting the composition of undiagnosed cases.

### Statistical analysis

We calculated diabetes prevalence by sex and age group, taking into account complex survey design and survey sample weights when relevant. We excluded age–sex groups with fewer than 25 participants when calculating prevalence because the sampling error of estimated prevalence can bias the associations between prevalences based on different definitions. Some surveys had measured HbA_1c_ or FPG in all participants, but had not measured 2hOGTT among people with diagnosed diabetes. These previously diagnosed participants were included in calculation of diabetes prevalence because their exclusion would underestimate diabetes prevalence. Furthermore, some surveys measured 2hOGTT in only a subset of people without history of diabetes diagnosis, generally for logistical or cost reasons. Simply combining these participants with previously diagnosed participants might overestimate diabetes prevalence based on 2hOGTT. To account for these missing measurements, and to avoid overestimation of diabetes prevalence, we recalculated the survey sample weights for these participants as the original sample weights divided by weighted proportion of non-diabetic participants with data. This approach is similar to that used in the US National Health and Nutrition Examination Survey for their 2hOGTT sample weights.^72^ A similar approach was taken in a few surveys that had measured HbA_1c_ in all participants, but had not measured FPG among people diagnosed with diabetes.

We compared graphically the prevalences of diabetes using different definitions. We also did regression analyses of the relation between diabetes defined (1) on the basis of FPG-or-2hOGTT versus on the basis of FPG only and (2) on the basis of HbA_1c_ versus on the basis of FPG. We did not do a regression for diabetes prevalence based on HbA_1c_ versus prevalence based on FPG-or-2hOGTT because very few surveys had data for both 2hOGTT and HbA_1c_, leading to unstable regression coefficients. We probit-transformed diabetes prevalence because it provided better fit to the data and it avoids predicting prevalences that are less than 0 or greater than 1. We considered regression models with alternative covariates and specifications, and chose the best model using the Bayesian information criterion, which measures the relative goodness of fit of a model; it rewards how well the model fits the data but discourages overfitting.^73^ The regressions included age (mean age of each age–sex group); the years over which each survey collected data (as the midyear of the period of data collection; appendix); national income (natural logarithm of per person gross domestic product) in the survey country and year; whether the study was representative of a national, subnational, or community population; and mean BMI for each age–sex group. Sex was excluded from the regressions on the basis of the Bayesian information criterion. The regression of diabetes prevalence based on HbA_1c_ against diabetes prevalence based on FPG, for which there were more data, also included terms for geographical region as random effects on the basis of Bayesian information criterion; these random effects account for differences in the relationship by region. Two regions consisted of high-income countries, as in previous global analyses^5^, ^74^—high-income Asia Pacific (consisting of Japan, Singapore, and South Korea) and high-income western countries (consisting of countries in Australasia, North America, and western Europe). The other countries were divided based on their geography into central and eastern Europe; central Asia, Middle East and north Africa; east and southeast Asia; south Asia; Latin America and the Caribbean; and sub-Saharan Africa.

We plotted the residuals of the regression models against the main independent variable (probit-transformed FPG-based prevalence), and found no evidence of heteroscedasticity in the residuals. We also report the univariate and semipartial R^2^ for each of the variables in the regression model. Univariate R^2^ measures how much of the variance is explained by each independent variable. Semipartial R^2^ measures the contribution of each variable to the total explained variance, conditional on the presence of the other model variables.^75^

We calculated sensitivity and specificity of diagnosis separately in each survey, and then pooled the sensitivities and specificities across surveys with a random-effects model.^76^ We examined the sources of heterogeneity in sensitivity and specificity with metaregressions and a-priori selected study characteristics: mean age, proportion of male participants, midyear of study data collection period; sample size; prevalence of undiagnosed diabetes in the survey; whether the survey was representative of a national, subnational, or community population; geographical region; national income in the survey country and year; and mean haemoglobin concentration in the survey country and year. We did the analyses with Stata (version 12.2) and R (version 3.0.3).

### Role of the funding source

The funders had no role in study design, data collection, analysis, or interpretation, or writing of the report. SF, YL, and BZ had full access to all the data. ME was responsible for submitting the Article for publication.

## Results

**Figure 1:**
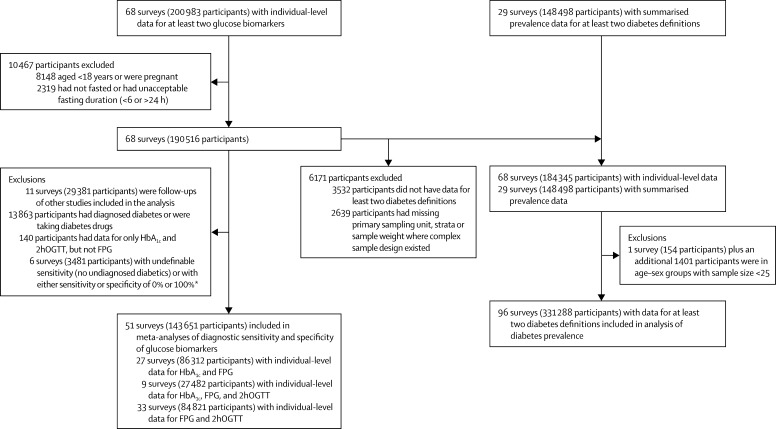
Study and data inclusion FPG=fasting plasma glucose. 2hOGTT=2-h oral glucose tolerance test. *The meta-analyses used inverse of variance as survey weights; sensitivity or specificity of either 0% or 100% would make the corresponding variance zero, and therefore the inverse of variance infinite.

After exclusions, we included 96 population-based health examination surveys of 331 288 participants (figure 1). 46 surveys were from Australia, USA, and western Europe; 18 from east and southeast Asia; ten from Latin America and the Caribbean; seven from Oceania; six from sub-Saharan Africa; five from south Asia; three from the Middle East and north Africa; and one from central and eastern Europe. All 96 studies measured FPG; 47 also measured 2hOGTT and 63 measured HbA_1c_ (appendix). 14 of these studies measured all three biomarkers. All but three studies of the 47 studies used for comparing prevalence based on FPG alone versus based on FPG-or-2hOGTT measured FPG in a laboratory; two of the remaining studies used a portable unit, and we did not have information for the remaining study. All studies measured 2hOGTT in a laboratory. All but one of the 63 studies used for comparing glucose-based and HbA_1c_-based prevalences measured glucose in a laboratory; the remaining study measured FPG with a portable unit. An enzymatic method was used to measure FPG in 65 of the 92 studies that had measured FPG in a laboratory, but we had no information for the remaining 27 studies. In all 63 studies, HbA_1c_ was measured in a laboratory; in 40 of these studies, the measurements were done by chromatography or immunoassay. No information was available for the remaining 23. Such a dominance of laboratory-based measurements prevented us from assessing the role of measurement method as a source of variation because laboratory-based methods are equally acceptable, especially for glucose.^77^

**Figure 2:**
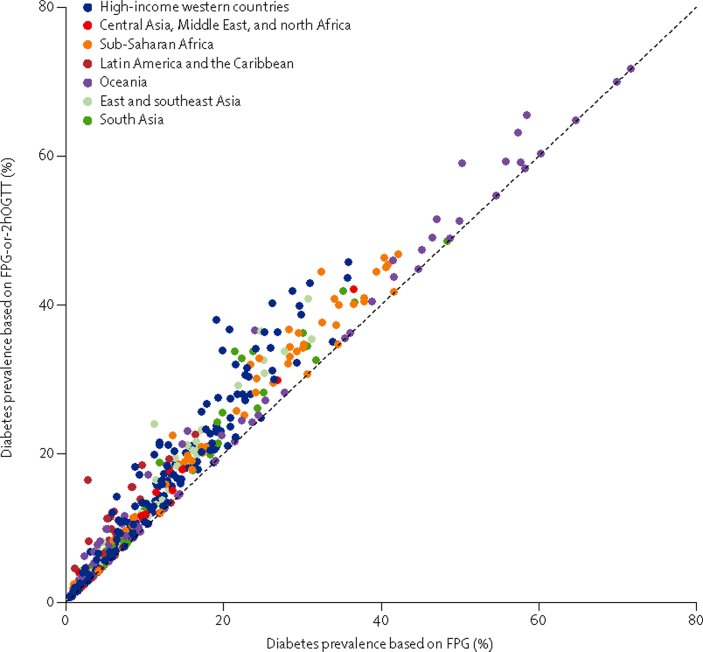
Prevalence of diabetes defined by FPG-or-2hOGTT versus by FPG only FPG-or-2hOGTT definition was FPG 7·0 mmol/L or more, or 2hOGTT 11·1 mmol/L or more, or history of diabetes or using insulin or oral hypoglycaemic drugs. FPG only definition was FPG 7·0 mmol/L or more, or history of diabetes or using insulin or oral hypoglycaemic drugs. Each point shows one age–sex group in one survey. Table 1 shows the relation summarised as regression coefficients. FPG=fasting plasma glucose. 2hOGTT=2-h oral glucose tolerance test.

Diabetes prevalence ranged from 0% in people younger than 40 years of age in some surveys to about 70% in middle-aged and older adults in Nauru (figure 2). Prevalence of diabetes based on FPG alone was lower than that based on FPG-or-2hOGTT, by 2–6 percentage points at different prevalence levels, although prevalences estimated using these two glucose-based measures were highly correlated (r=0·98; figure 2). Table 1, Table 2 show results of the regression analyses. After accounting for prevalence based on FPG, prevalence based on FPG-or-2hOGTT increased with age—ie, prevalence based on FPG-or-2hOGTT rose more sharply with age than did prevalence based on FPG only.^65^, ^80^, ^81^

**Figure 3:**
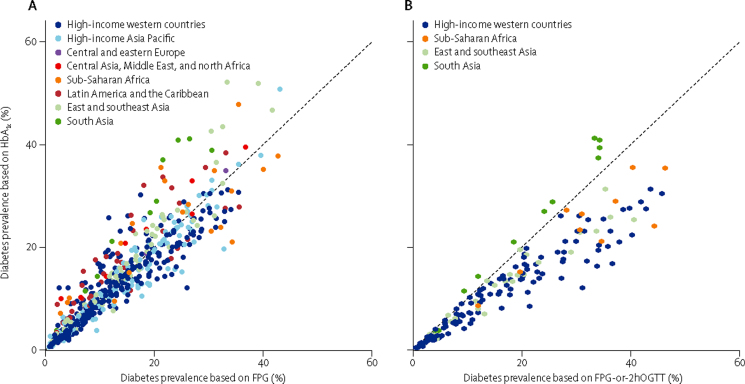
Prevalence of diabetes defined by HbA_1c_ only versus prevalence defined by (A) FPG only, and (B) FPG-or-2hOGTT HbA_1c_ definition was HbA_1c_ 6·5% or more, or history of diabetes, or using insulin or oral hypoglycaemic drugs. FPG only definition was FPG 7·0 mmol/L or more, or history of diabetes or using insulin or oral hypoglycaemic drugs. FPG-or-2hOGTT definition was FPG 7·0 mmol/L or more, or 2hOGTT 11·1 mmol/L or more, or history of diabetes or using insulin or oral hypoglycaemic drugs. Each point shows one age–sex group in one survey. Table 2 shows the relations summarised as regression coefficients. FPG=fasting plasma glucose. 2hOGTT=2-h oral glucose tolerance test.

HbA_1c_-based prevalences were lower than those based on FPG for 42·8% of age–sex–survey groups and higher in another 41·6%; in the other 15·6%, the two definitions gave similar prevalences (figure 3). In the regression analysis, prevalence based on HbA_1c_ was on average slightly lower than prevalence based on FPG (table 2). The most important determinant of variation between these two prevalences was age, with some effect from national income, mean BMI, year of survey, and whether the survey was representative of a national, subnational, or community population. After accounting for prevalence based on FPG, prevalence based on HbA_1c_ increased with age, national income, mean BMI, and the year of survey. After accounting for prevalence based on FPG, HbA_1c_-based prevalence was higher in south Asia than in other regions, and was lower in high-income regions than in other regions (appendix).

Diabetes defined as HbA_1c_ of 6·5% or more had a pooled sensitivity of 52·8% (95% CI 51·3–54·3) compared with a definition of FPG of 7·0 mmol/L or more for diagnosing participants without a previous diagnosis of diabetes. This finding suggests that 47·2% of participants without a previous diagnosis of diabetes who would be considered to have diabetes based on their FPG concentration would not be considered to have diabetes with an HbA_1c_ test (table 3). The sensitivity of HbA_1c_ varied substantially across studies (*I*^2^ of 97·6%), ranging from 13·0% to 93·2% (appendix pp 11–12). HbA_1c_ had even lower sensitivity when compared with defining diabetes based on FPG-or-2hOGTT (30·5%, 95% CI 28·7–32·3). None of the preselected study-level characteristics explained the heterogeneity in the sensitivity of HbA_1c_ versus FPG (all p values >0·06; table 4). Pooled specificity of HbA_1c_ was 99·74% (95% CI 99·71–99·78) relative to FPG and 99·69% (99·63–99·76) relative to FPG-or-2hOGTT, suggesting few false positives compared with glucose-based definitions.

Lowering the threshold for diabetes by HbA_1c_ from 6·5% to 6·3% (a cutoff suggested by some studies^49^, ^50^) increased sensitivity compared with the FPG-based definition from 52·8% to 64·3% while maintaining a high specificity at 99·53%. Lowering it further to 6·1% increased sensitivity to 72·8% but the specificity would drop to 99·08%, resulting in more false positives. Follow-up studies are needed to establish how these cutoffs predict complications and sequelae in newly diagnosed patients.^83^, ^84^

## Discussion

In this large international pooled analysis of population-based health examination surveys, we found that the use of different biomarkers and definitions for diabetes can lead to different estimates of population prevalence of diabetes, with the highest prevalence when diabetes is defined on the basis of FPG-or-2hOGTT and the lowest when based on HbA_1c_ alone. For example, at an FPG-based prevalence of 10%, similar to the age-standardised global prevalence of diabetes in adults aged 25 years and older in 2008,^1^ prevalence based on FPG-or-2hOGTT would be about 13% according to the relation in figure 2. The variation across studies in the relation between glucose-based and HbA_1c_-based prevalences was partly related to age, followed by national income, mean BMI, the year of survey, and whether the survey population was national, subnational, or from specific communities. The reasons for additional regional effects—higher HbA_1c_-based prevalence in south Asia and lower prevalence in high-income regions than in other regions after accounting for prevalence based on FPG—are unknown, but they might be a result of true physiological differences; for example, related to red blood cell turnover (itself related to anaemia and iron status), which affects HbA_1c_, or related to glucose dysregulation during fasting and non-fasting which are captured by HbA_1c_.^85^ Establishing these reasons requires multicentre studies with consistent methods and protocols and data for phenotypical factors that might affect the relation between glucose and HbA_1c_. For now, they are unexplained empirical results that should be taken into account when using surveys from different regions.

Similarly, different definitions identified different people without a previous diagnosis as having diabetes. Specifically, use of an HbA_1c_-based definition would not identify almost half of the undiagnosed cases that could be detected with a FPG test, and more than three-quarters of undiagnosed cases that would be detected by FPG and 2hOGTT combined, but it would lead to few false positives compared with glucose-based definitions. Inversely, using a glucose-based test alone would not identify some people who would be considered as having diabetes with HbA_1c_.

Our results, based on a large number of surveys from different regions, are consistent with previous smaller studies that compared different diabetes definitions. Diabetes prevalence based on FPG-or-2hOGTT was higher than prevalence based on FPG alone by 18% in an analysis of 13 European cohorts and by 6% in an analysis of 11 Asian cohorts.^63^, ^64^ A previous comparison of diabetes prevalence across six studies, including two analysed here, reported that diagnostic sensitivity for HbA_1c_ compared with 2hOGTT ranged from 17% to 78%,^67^ which is consistent with the results of our analysis. However, this study also found surprisingly low specificities for HbA_1c_ compared with ours.^67^ Other single-cohort studies also generally reported low but variable sensitivities and high specificities for HbA_1c_ relative to blood glucose. Several studies^86^, ^87^, ^88^, ^89^ assessed the optimal cutoff for HbA_1c_ in different populations and all reported values lower than 6·5%, which is consistent with our finding that lowering the threshold would increase sensitivity while preserving high specificity. One small study^90^ examined the effect of anaemia on diagnostic accuracy of HbA_1c_ and reported higher sensitivity (than with FPG) in patients with anaemia, which is consistent with our results.

Our analysis, which focused on questions that are relevant for population-based surveillance of diabetes and monitoring treatment coverage, has several strengths. We pooled data from a large number of population-based surveys from different world regions, thereby increasing both the precision of our estimates and their generalisability compared with analyses of one or a small number of cohorts. We used consistent eligibility and inclusion criteria, and assessed whether the surveys met these criteria. In particular, we only used surveys that had rigorous protocols for fasting duration and for OGTT. Furthermore, most surveys measured glucose and HbA_1c_ in a laboratory. We also assessed the sources of heterogeneity in how diagnostic criteria compare across surveys, which could not be done in previous analyses because they included few surveys.

Our results should be interpreted with some limitations in mind. We had few studies from some regions including sub-Saharan Africa, south Asia, the Middle East and north Africa, and central and eastern Europe. We analysed the surveys with consistent methods but surveys might have differed in details such as the exact limit for fasting duration beyond the 6-h limit imposed by us. Because HbA_1c_ measurement has changed over time,^91^, ^92^, ^93^, ^94^, ^95^, ^96^, ^97^, ^98^, ^99^ and to minimise the use of non-standard assays, we did not include any HbA_1c_ data from before the year 2000.^71^ Despite this exclusion, and the fact that all of our surveys had measured HbA_1c_ in a laboratory, HbA_1c_ measurements can vary between laboratories and instruments,^100^ about which we did not have complete data. For the same reason, we could not standardise the HbA_1c_ data to account for different assays and instruments used in measurement. Nutritional status—especially iron deficiency—anaemia, malaria and other parasitic diseases, living at high altitudes, and high prevalence of haemoglobinopathies can affect HbA_1c_,^101^ but could not be assessed as a source of heterogeneity beyond their effects through mean haemoglobin concentration. Similarly, data for glucose can be affected by unrecorded factors such as inaccurate information about fasting, fluctuations in diet and physical activity in days before measurement, and how samples were handled, including time between drawing blood and laboratory analysis and the type of tube used for collecting and storing blood.

Although we assessed the role of geographical region, we did not have data for the ethnic composition of participants in each survey. By their nature, health examination surveys used for population-based surveillance use a single measurement for each participant, whereas diagnosis in a clinical setting might repeat the measurements based on the first test. The use of a single test is affected by within-individual and even within-laboratory variation, and could lead to misclassification of some individuals.^99^ Finally, we did not have longitudinal follow-up data to assess sensitivity and specificity for diagnosis using one definition (or one cutoff value of HbA_1c_) compared to another or for development of diabetes complications and sequelae that contribute the bulk of the public health burden of diabetes. Such data are not available in population-based surveys because surveys are typically cross-sectional.

There is no gold standard definition that captures the phenotypic complexity of diabetes and the risk of its microvascular and macrovascular complications, although 2hOGTT is often treated as the most reliable test.^15^, ^102^, ^103^ In clinical practice, physicians follow an analytical process to diagnose diabetes, in which different sequences of glucose biomarkers are used depending on factors such as a patient's age and symptoms; those with high levels of one biomarker (eg, HbA_1c_) might be asked to have additional measurements of the same or a different biomarker, and be monitored over time to decide on the best course of treatment. The process might vary from patient to patient to account for their unique characteristics, and might further vary from physician to physician based on available infrastructure and medical resources. In surveillance using population-based surveys, which provides evidence for policies and programmes related to whole populations, repeated measurements are virtually impossible. Therefore, considerations about diabetes definition and diagnosis are different from those of clinical practice, and the emphasis is on comparability of definitions over time and across populations. Our results provide much needed empirical evidence for planning global surveillance of diabetes and coverage of its interventions. Specifically, despite its relative ease of use, using HbA_1c_ alone in health surveys might miss some previously undiagnosed people who would be considered as having diabetes using a glucose-based test, and thus could benefit from lifestyle and treatment interventions. Even so, 2hOGTT is difficult to measure even in a clinical setting, let alone in population-based surveys. Of 493 worldwide population-based diabetes data sources between 1975 and 2014 in the NCD-RisC databases, 448 had measured FPG but only 59 had measured 2hOGTT; 33% of surveys before 1990 had 2hOGTT and only 11% did after 1990. Therefore, a strategy for consistent and comparable surveillance is to use FPG in population-based surveys, be it national or multicountry survey programmes such as the WHO STEPS surveys, and define diabetes based on FPG. Data such as those in figure 2 and table 1 can then be used to relate prevalences based on FPG to those based on FPG-or-2hOGTT. The use of HbA_1c_ in surveillance requires further consideration of how it predicts and helps prevent diabetes complications and sequelae. When HbA_1c_ is used, FPG should ideally also be measured in a subsample of participants to provide information about how the two tests relate.

Correspondence to: Prof Majid Ezzati, Imperial College London, London W2 1PG, UK majid.ezzati@imperial.ac.uk

## Figures and Tables

**Table 1:** Regression coefficients for the relation between probit-transformed prevalence of diabetes based on FPG-or-2hOGTT versus diabetes based on FPG only

		**Coefficient (95% CI)**	**p value**	**Univariate R**^2^*	**Semipartial R**^2^†
Intercept		0·135 (−0·020 to 0·290)	0·0872	NA	NA
Probit-transformed prevalence of diabetes based on FPG		0·903 (0·880 to 0·927)	<0·0001	0·963	0·368
Mean age of age–sex group (per 10 years older)		0·048 (0·039 to 0·056)	<0·0001	0·444	0·008
Study midyear (per one more recent year since 1976)		−0·001 (−0·002 to 0·000)	0·1643	0·003	<0·001
Natural logarithm of per person gross domestic product		−0·033 (−0·046 to −0·019)	<0·0001	0·004	0·001
Mean BMI		0·000 (−0·004 to 0·004)	0·9057	0·092	<0·001
Study representativeness		..	..	0·021	0·001
	National	Reference	..	..	..
	Subnational	−0·031 (−0·070 to 0·008)	0·1141	..	..
	Community	−0·070 (−0·101 to −0·039)	<0·0001	..	..

FPG=fasting plasma glucose. 2hOGTT=2-h oral glucose tolerance test.

* Calculated by regressing against each independent variable alone; equals the square of the correlation coefficient.

† Shows how much R^2^ decreases if that independent variable is removed from the full model; the overall R^2^ for the model was 0·973.

**Table 2:** Regression coefficients for the association between probit-transformed prevalence of diabetes based on HbA_1c_ and probit-transformed prevalence based on FPG

		**Coefficient (95% CI)**	p value*	**Univariate R^2^**†	**Semipartial R^2^**‡
Intercept		−1·761 (−2·229 to −1·266)	<0·0001	NA	NA
Probit-transformed prevalence of diabetes based on FPG		0·799 (0·763 to 0·835)	<0·0001	0·915	0·075
Mean age of age–sex group (per 10 years older)		0·052 (0·042 to 0·062)	<0·0001	0·601	0·011
Study midyear (per one more recent year since 2000)		0·012 (0·009 to 0·015)	<0·0001	0·014	0·006
Natural logarithm of per person gross domestic product		0·076 (0·035 to 0·114)	0·0001	0·052	0·003
Mean BMI		0·018 (0·010 to 0·027)	<0·0001	0·022	0·002
Study representativeness		..	..	0·013	0·004
	National	Reference	..	..	..
	Subnational	−0·004 (−0·047 to 0·040)	0·8758	..	..
	Community	0·090 (0·060 to 0·119)	<0·0001	..	..

The appendix shows regional random effects. FPG=fasting plasma glucose.

* p values using likelihood ratio test, which compares the likelihood of the models with and without the variable of interest.^78^

† Calculated by regressing against each independent variable alone, without the regional random effect; equals the square of the correlation coefficient.

‡ Is the decrease of R^2^ if one of the independent variables is removed from the full model; however, traditional R^2^ is not clearly defined for mixed-effect models, we have used the conditional R^2^ that describes the proportion of variance explained by both fixed and random factors.^79^ The overall conditional R^2^ for the model was 0·949.

**Table 3:** Pooled sensitivity and specificity of diabetes diagnosis using different definitions among participants without diagnosed diabetes

	**Number of surveys**	**Sensitivity**		**Specificity**	
		(%; 95% CI)	*I*^2^	(%; 95% CI)	*I*^2^
HbA_1c_*vs* FPG	27	52·82 (51·33–54·30)	97·6%	99·74 (99·71–99·78)	98·2%
HbA_1c_*vs* 2hOGTT	9	37·16 (35·05–39·28)	97·6%	99·84 (99·79–99·89)	97·3%
HbA_1c_*vs* FPG-or-2hOGTT	9	30·46 (28·66–32·25)	97·9%	99·69 (99·63–99·76)	98·0%
FPG *vs* 2hOGTT	33	54·42 (53·26–55·57)	96·9%	98·90 (98·83–98·97)	94·4%

The appendix shows detailed results of these meta-analyses. Diabetes was defined as HbA_1c_ ≥6·5%, FPG ≥7·0 mmol/L, and 2hOGTT ≥11·1 mmol/L. FPG=fasting plasma glucose. 2hOGTT=2-h oral glucose tolerance test.

**Table 4:** Univariate metaregression coefficients for sensitivity of HbA_1c_ versus FPG in participants without diagnosed diabetes

		**Mean difference in sensitivity (percentage points; 95% CI)**	**p value**
Mean age (per 10 years older)		−4·1 (−12·7 to 4·5)	0·3361
Percent male participants (per 10% more male)		4·6 (−9·0 to 18·2)	0·4901
Study midyear (per one more recent year)		1·2 (−0·9 to 3·2)	0·2566
Region		..	0·2097
	High-income western countries	Reference group	..
	East, south, and southeast Asia	21·0 (−0·3 to 42·2)	..
	Latin America and the Caribbean	8·5 (−17·9 to 34·9)	..
	Sub-Saharan Africa	17·6 (−14·1 to 49·2)	..
Study representativeness		..	0·0915
	National	Reference group	..
	Subnational	1·7 (−28·6 to 31·9)	..
	Community	21·4 (2·1 to 40·8)	..
Prevalence of undiagnosed diabetes (percentage point higher undiagnosed diabetes)		−0·7 (−4·0 to 2·6)	0·6780
Sample size (per 1000 participants without diagnosed diabetes)		−1·6 (−4·6 to 1·4)	0·2730
Natural logarithm of per person gross domestic product		−6·5 (−17·6 to 4·6)	0·2410
Mean haemoglobin (per g/L)*		−2·0 (−4·1 to 0·2)	0·0677

We used a HbA_1c_ definition of 6·5% or more and a FPG definition of 7·0 mmol/L or more. FPG=fasting plasma glucose.

* Reliable mean haemoglobin data were available only for women of child-bearing age.^82^ The national mean for each country-year was used for both men and women; restricting the analysis to women led to similar results, with a mean difference of −2·1 (−4·5 to 0·3, p=0·0929).

## Supplementary Material

Supplementary appendix

Supplementary audio How do different methods for diagnosing diabetes affect estimates of prevalence and how is this likely to affect global estimates of this disease?
