# Supplementary material for: Effects of diabetes definition on global surveillance of diabetes prevalence and diagnosis: a pooled analysis of 96 population-based studies with 331 288 participants
Source: Lancet Diabetes Endocrinol. 2015 Aug;3(8):624–37. doi: 10.1016/S2213-8587(15)00129-1 (PMC4673089; doi:10.1016/S2213-8587(15)00129-1)
Supplement: Supplementary appendix [file mmc1.pdf]

THE LANCET Diabetes & Endocrinology

Supplementary appendix

This appendix formed part of the original submission and has been peer reviewed. We post it as supplied by the authors.

Supplement to: NCD Risk Factor Collaboration (NCD-RisC). Effects of diabetes definition on global surveillance of diabetes prevalence and diagnosis: a pooled analysis of 96 population-based studies with 331 288 participants. *Lancet Diabetes Endocrinol* 2015; published online June 22. http://dx.doi.org/10.1016/S2213-8587(15)00129-1.

Webmaterials

Table of Contents

Webtable 1: Population-based surveys with individual-level data 1

Webtable 2: Population-based surveys with summarised prevalence data 6

Webfigure 1: Regional random effects in the regression of diabetes prevalence defined based on HbA_{1c} ($HbA_{1c} \geq$ 6.5% or history of diabetes or using insulin or oral hypoglycaemic agents) against diabetes prevalence defined based on FPG (FPG $\geq$ 7.0 mmol/L or history of diabetes or using insulin or oral hypoglycaemic agents) 9

Webfigure 2: Random effect meta-analysis of diabetes diagnostic sensitivity of $HbA_{1c} \geq$ 6.5% vs. FPG $\geq$ 7.0 mmol/L in previously-undiagnosed participants 11

Webfigure 3: Random effect meta-analysis of diabetes diagnostic specificity of $HbA_{1c} \geq$ 6.5% vs. FPG $\geq$ 7.0 mmol/L in previously-undiagnosed participants 13

Webfigure 4: Random effect meta-analysis of diabetes diagnostic sensitivity of $HbA_{1c} \geq$ 6.5% vs. 2hOGTT $\geq$ 11.1 mmol/L in previously-undiagnosed participants 15

Webfigure 5: Random effect meta-analysis of diabetes diagnostic specificity of $HbA_{1c} \geq$ 6.5% vs. 2hOGTT $\geq$ 11.1 mmol/L in previously-undiagnosed participants 17

Webfigure 6: Random effect meta-analysis of diabetes diagnostic sensitivity of $HbA_{1c} \geq$ 6.5% vs. FPG $\geq$ 7.0 mmol/L or 2hOGTT $\geq$ 11.1 mmol/L in previously-undiagnosed participants 19

Webfigure 7: Random effect meta-analysis of diabetes diagnostic specificity of $HbA_{1c} \geq$ 6.5% vs. FPG $\geq$ 7.0 mmol/L or 2hOGTT $\geq$ 11.1 mmol/L in previously-undiagnosed participants 21

Webfigure 8: Random effect meta-analysis of diabetes diagnostic sensitivity of FPG $\geq$ 7.0 mmol/L vs. 2hOGTT $\geq$ 11.1 mmol/L in previously-undiagnosed participants 23

Webfigure 9: Random effect meta-analysis of diabetes diagnostic specificity of FPG $\geq$ 7.0 mmol/L vs. 2hOGTT $\geq$ 11.1 mmol/L in previously-undiagnosed participants 25

References 27

Webtable 1: Population-based surveys with individual-level data

Survey name or citation	Country	Survey year(s)	Participants ≥ 18 years	Mean age (SD)	% Male	Level of representative-ness	Rural, urban or both	Biomarkers available	Glucose measurement	HbA_{1c} measurement
Australian Diabetes Obesity and Lifestyle Study (AusDiab)	Australia	1999-2000	11,173	51.6 (14.4)	45.1%	National	Both	FPG, 2hOGTT, HbA_{1c}	Lab	Lab
Australian Diabetes Obesity and Lifestyle Study (AusDiab)	Australia	2004-2005	6,297	56.5 (12.7)	45.3%	National	Both	FPG, 2hOGTT, HbA_{1c}	Lab	Lab
Australian Diabetes Obesity and Lifestyle Study (AusDiab)	Australia	2011-2012	4,481	60.9 (11.2)	44.7%	National	Both	FPG, 2hOGTT, HbA_{1c}	Lab	Lab
North West Adelaide Health Study (NWAHS)	Australia	1999-2003	3,979	50.5 (16.4)	47.5%	Community	Urban	FPG, HbA_{1c}	Lab	Lab
North West Adelaide Health Study (NWAHS)	Australia	2004-2006	3,163	55.1 (15.5)	47.4%	Community	Urban	FPG, HbA_{1c}	Lab	Lab
North West Adelaide Health Study (NWAHS)	Australia	2008-2010	2,420	57.9 (14.3)	47.1%	Community	Urban	FPG, HbA_{1c}	Lab	Lab
The Central America Diabetes Initiative (CAMDI) Survey	Belize	2004-2005	1,306	45.7 (17.4)	38.9%	National	Both	FPG, 2hOGTT	Lab	-
Baependi Heart Study	Brazil	2010-2013	1,233	44.6 (16.5)	40.6%	Community	Rural	FPG, HbA_{1c}	Lab	Lab
China Health and Nutrition Study (CHNS)	China	2009	8,496	50.5 (15.0)	47.0%	National	Both	FPG, HbA_{1c}	Lab	Lab
Hong Kong Cardiovascular Risk Factor Prevalence Study	China (Hong Kong SAR)	1995-1996	2,762	45.9 (12.9)	49.2%	National	Both	FPG, 2hOGTT	Lab	-
The Central America Diabetes Initiative (CAMDI) Survey	Costa Rica	2004	974	45.3 (17.1)	32.9%	Community	Urban	FPG, 2hOGTT	Lab	-
Costa Rican Longevity and Healthy Aging Study (CRELES), Pre-1945 Cohort Wave 1	Costa Rica	2004-2006	2,587	76.2 (10.2)	45.3%	National	Both	FPG, HbA_{1c}	Lab	Lab
Costa Rican Longevity and Healthy Aging Study (CRELES), Pre-1945 Cohort Wave 2	Costa Rica	2006-2008	2,218	77.0 (9.6)	45.5%	National	Both	FPG, HbA_{1c}	Lab	Lab
Young Finns Study (YFS), Rural	Finland	2011	786	42.0 (4.9)	46.3%	National	rural	FPG, HbA_{1c}	Lab	Lab
Young Finns Study (YFS), Urban	Finland	2011	1,121	41.9 (5.1)	44.2%	National	Urban	FPG, HbA_{1c}	Lab	Lab
The Three City Study (3C Study)	France	2008-2010	693	82.3 (4.4)	36.1%	Community	Urban	FPG, HbA_{1c}	Lab	Lab
Population Health Survey in Greenland	Greenland	2005-2010	3,046	44.5 (14.7)	43.9%	National	Both	FPG, 2hOGTT, HbA_{1c}	Lab	Lab
The Central America Diabetes Initiative (CAMDI) Survey	Guatemala	2001-2002	848	40.5 (15.4)	33.6%	Community	Urban	FPG, 2hOGTT	Lab	-
The Central America Diabetes Initiative (CAMDI) Survey	Honduras	2003-2004	1,179	40.6 (15.6)	35.5%	Community	Urban	FPG, 2hOGTT	Lab	-
New Delhi Birth Cohort, Follow-up Phase 1	India	1999-2002	1,434	28.7 (1.4)	59.0%	Community	Urban	FPG, 2hOGTT	Lab	-
New Delhi Birth Cohort, Follow-up Phase 2	India	2006-2009	1,075	35.6 (1.1)	59.5%	Community	Urban	FPG, 2hOGTT	Lab	-
Chennai Urban Rural Epidemiology Study (CURES)	India	2001-2004	2,349	39.6 (12.9)	46.7%	Community	Urban	FPG, 2hOGTT, HbA_{1c}	Lab	Lab

Survey name or citation	Country	Survey year(s)	Participants ≥ 18 years	Mean age (SD)	% Male	Level of representative -ness	Rural, urban or both	Biomarkers available	Glucose measurement	HbA_{1c} measurement
High Prevalence of Diabetes and Cardiovascular Risk Factors Associated with Urbanization in India. Diabetes Care. 2008;31:893-8.	India	2006	7,057	38.0 (12.1)	47.0%	Community	Both	FPG, 2hOGTT	Lab	-
Bruneck Study	Italy	1990	908	58.8 (11.4)	51.1%	Community	Rural	FPG, 2hOGTT	Lab	-
Bruneck Study	Italy	1995	775	62.6 (11.1)	50.6%	Community	Rural	FPG, 2hOGTT	Lab	-
Bruneck Study	Italy	2000	692	66.0 (10.3)	47.8%	Community	Rural	FPG, HbA_{1c}	Lab	Lab
Bruneck Study	Italy	2005	568	69.1 (9.5)	46.3%	Community	Rural	FPG, HbA_{1c}	Lab	Lab
Bruneck Study	Italy	2010	484	72.5 (8.5)	46.5%	Community	Rural	FPG, HbA_{1c}	Lab	Lab
Epidemiological Survey of Kiribati, Rural	Kiribati	1981	970	41.7 (15.8)	47.3%	Subnational	Rural	FPG, 2hOGTT	Lab	-
Epidemiological Survey of Kiribati, Urban	Kiribati	1981	1,720	35.9 (12.4)	50.6%	Subnational	Urban	FPG, 2hOGTT	Lab	-
Metabolic Syndrome Study in Malaysia	Malaysia	2008	4,091	48.1 (14.5)	34.9%	National	Both	FPG, 2hOGTT, HbA_{1c}	Lab	Lab
Mauritius Noncommunicable Disease Survey	Mauritius	1987	4,960	43.4 (13.2)	46.9%	National	Both	FPG, 2hOGTT	Lab	-
Mauritius Noncommunicable Disease Survey	Mauritius	1992	6,453	45.6 (12.1)	46.2%	National	Both	FPG, 2hOGTT	Lab	-
Mauritius Noncommunicable Disease Survey	Mauritius	1998	5,355	48.9 (11.4)	44.3%	National	Both	FPG, 2hOGTT	Lab	-
Mauritius Noncommunicable Disease Survey	Mauritius	2009	6,080	45.9 (13.6)	45.7%	National	Both	FPG, 2hOGTT	Lab	-
Trends in the Prevalence and Incidence of Non-insulin-dependent Diabetes Mellitus and Impaired Glucose Tolerance	Nauru	1975-1976	425	36.1 (13.5)	48.5%	Subnational	Both	FPG, 2hOGTT	Lab	-
Trends in the Prevalence and Incidence of Non-insulin-dependent Diabetes Mellitus and Impaired Glucose Tolerance	Nauru	1982	1,393	36.5 (13.4)	47.5%	National	Both	FPG, 2hOGTT	unknown	-
Trends in the Prevalence and Incidence of Non-insulin-dependent Diabetes Mellitus and Impaired Glucose Tolerance	Nauru	1987	1,072	39.3 (12.8)	45.3%	National	Both	FPG, 2hOGTT	Lab	-
Trends in the Prevalence and Incidence of Non-insulin-dependent Diabetes Mellitus and Impaired Glucose Tolerance	Nauru	1994	1,364	39.0 (11.3)	47.1%	National	Both	FPG, 2hOGTT	Lab	-
The Central America Diabetes Initiative (CAMDI) Survey	Nicaragua	2003-2004	1,552	40.2 (14.8)	46.0%	Community	Urban	FPG, 2hOGTT	Lab	-
Survey in Ramallah District, Rural *	Occupied Palestinian Territory	1996-1998	606	37.9 (12.7)	33.5%	Community	Rural	FPG, 2hOGTT	Portable for FPG Lab for 2hOGTT	-
Survey in Ramallah District, Urban *	Occupied Palestinian Territory	1996-1998	637	38.6 (12.7)	28.6%	Community	Urban	FPG, 2hOGTT	Portable for FPG Lab for 2hOGTT	-

Survey name or citation	Country	Survey year(s)	Participants ≥ 18 years	Mean age (SD)	% Male	Level of representative -ness	Rural, urban or both	Biomarkers available	Glucose measurement	HbA_{1c} measurement
Peru Migrant Study	Peru	2007-2008	848	50.6 (10.9)	48.0%	Community	Both	FPG, HbA_{1c}	Lab	Lab
CRONICAS Cohort Study	Peru	2009-2012	3,114	55.2 (12.7)	48.7%	Subnational	Both	FPG, HbA_{1c}	Lab	Lab
Seychelles Heart Survey IV	Seychelles	2014	1,212	45.8 (11.1)	43.4%	National	Both	FPG, HbA_{1c}	Lab	Lab
Cape Town Bellville South Cohort Study, Baseline Evaluation I	South Africa	2008-2009	924	54.3 (14.7)	23.6%	Community	Urban	FPG, 2hOGTT, HbA_{1c}	Lab	Lab
Korea National Health and Nutrition Examination Survey	South Korea	2011	5,715	50.2 (16.6)	43.0%	National	Both	FPG, HbA_{1c}	Lab	Lab
Korea National Health and Nutrition Examination Survey	South Korea	2012	5,237	50.5 (16.6)	42.1%	National	Both	FPG, HbA_{1c}	Lab	Lab
Harmonizing Equation of Risk in Mediterranean countries Extremadura (The HERMEX study)	Spain	2007-2009	2,787	50.8 (14.4)	46.5%	Subnational	Both	FPG, HbA_{1c}	Lab	Lab
Study on Nutrition and Cardiovascular Risk in Spain	Spain	2008-2010	12,821	47.2 (16.7)	47.4%	National	Both	FPG, HbA_{1c}	Lab	Lab
Turkish Adult Risk Factor Study	Turkey	2012-2013	425	58.3 (10.1)	48.5%	National	Both	FPG, HbA_{1c}	Portable	Lab
The Funafuti Survey	Tuvalu	1976	415	38.2 (15.5)	49.4%	Subnational	Both	FPG, 2hOGTT	Lab	-
Hertfordshire Cohort Study	UK	1999-2004	2,758	65.7 (2.9)	52.5%	Subnational	Both	FPG, 2hOGTT	Lab	-
Health Survey for England	UK	2003	1,096	57.2 (14.0)	47.8%	National	Both	FPG, HbA_{1c}	Lab	Lab
English Longitudinal Study of Ageing, Wave 2	UK	2004-2005	3,701	63.8 (7.4)	46.5%	National	Both	FPG, HbA_{1c}	Lab	Lab
English Longitudinal Study of Ageing, Wave 4	UK	2008-2009	4,008	64.1 (7.5)	46.1%	National	Both	FPG, HbA_{1c}	Lab	Lab
English Longitudinal Study of Ageing, Wave 6	UK	2012-2013	3,465	65.8 (7.6)	44.0%	National	Both	FPG, HbA_{1c}	Lab	Lab
MRC National Survey of Health and Development (NSHD)	UK	2009	1,758	62.0 (0.0)	47.6%	National	Both	FPG, HbA_{1c}	Lab	Lab
United States National Health and Nutrition Examination Survey (US NHANES)	USA	1976-1980	3,808	48.8 (16.7)	47.2%	National	Both	FPG, 2hOGTT	Lab	-
United States National Health and Nutrition Examination Survey (US NHANES)	USA	1988-1994	3,310	56.7 (11.2)	48.7%	National	Both	FPG, 2hOGTT	Lab	-
United States National Health and Nutrition Examination Survey (US NHANES)	USA	1999-2000	1,943	47.6 (19.8)	50.0%	National	Both	FPG, HbA_{1c}	Lab	Lab
United States National Health and Nutrition Examination Survey (US NHANES)	USA	2001-2002	2,305	47.2 (20.0)	51.1%	National	Both	FPG, HbA_{1c}	Lab	Lab
United States National Health and Nutrition Examination Survey (US NHANES)	USA	2003-2004	2,111	47.9 (20.8)	50.6%	National	Both	FPG, HbA_{1c}	Lab	Lab
United States National Health and Nutrition Examination Survey (US NHANES)	USA	2005-2006	2,057	46.6 (20.1)	52.6%	National	Both	FPG, 2hOGTT, HbA_{1c}	Lab	Lab

**Survey name or citation**	**Country**	**Survey year(s)**	**Participants ≥ 18 years**	**Mean age (SD)**	**% Male**	**Level of representative-ness**	**Rural, urban or both**	**Biomarkers available**	**Glucose measurement**	**HbA_{1c} measurement**
United States National Health and Nutrition Examination Survey (US NHANES)	USA	2007-2008	2,450	49.5 (18.5)	49.7%	National	Both	FPG, 2hOGTT, HbA_{1c}	Lab	Lab
United States National Health and Nutrition Examination Survey (US NHANES)	USA	2009-2010	2,732	48.2 (18.5)	47.4%	National	Both	FPG, 2hOGTT, HbA_{1c}	Lab	Lab
United States National Health and Nutrition Examination Survey (US NHANES)	USA	2011-2012	2,409	47.3 (18.2)	50.4%	National	Both	FPG, 2hOGTT, HbA_{1c}	Lab	Lab

* Fasting glucose was measured in capillary whole blood, and was converted to plasma-equivalent by multiplying by 1.11.[1]

Webtable 2: Population-based surveys with summarised prevalence data

Survey name	Country	Survey year(s)	Participants ≥ 18 years	Level of representative-ness	Rural, urban or both	Biomarkers available	Glucose measurement	HbA_{1c} measurement
China Noncommunicable Disease Survey	China	2010-2011	97,630	National	Both	FPG, 2hOGTT, HbA_{1c}	Lab	Lab
Nutrition and Health of Aging Population in China	China	2011	2,241	Community	Both	FPG, HbA_{1c}	Lab	Lab
Kuopio Ischaemic Heart Disease Risk factor Study	Finland	1991-1993	517	National	Both	FPG, 2hOGTT	Lab	-
Kuopio Ischaemic Heart Disease Risk factor Study	Finland	1998-2001	1,135	National	Both	FPG, 2hOGTT	Lab	-
Kuopio Ischaemic Heart Disease Risk factor Study	Finland	2005-2008	1,703	National	Both	FPG, 2hOGTT	Lab	-
National Monitoring of Arterial Risk (MONA LISA) in Lille	France	2004-2006	1,378	Community	Urban	FPG, HbA_{1c}	Lab	Lab
National Monitoring of Arterial Risk (MONA LISA) in Bas-Rhin	France	2005-2007	1,459	Subnational	Both	FPG, HbA_{1c}	Lab	Lab
Etude Nationale Nutrition Sante	France	2006-2007	1,936	National	Both	FPG, HbA_{1c}	Lab	Lab
Enquête Littorale Souffle Air Biologie Environnement (ELISABET) Dunkerque	France	2011-2013	786	Community	Urban	FPG, HbA_{1c}	Lab	Lab
Enquête Littorale Souffle Air Biologie Environnement (ELISABET) Lille	France	2011-2013	1,322	Community	Urban	FPG, HbA_{1c}	Lab	Lab
KORA S4 Study: Kooperative Research in the Region of Augsburg Survey 4	Germany	1999-2001	1,641	Subnational	Both	FPG, 2hOGTT, HbA_{1c}	Lab	Lab
KORA F4 Study: Kooperative Research in the Region of Augsburg Follow-Up of Survey 4	Germany	2006-2008	2,829	Subnational	Both	FPG, 2hOGTT, HbA_{1c}	Lab	Lab
German Health Interview and Examination Survey for Adults 2008-2011 (DEGS1)	Germany	2008-2011	5,560	National	Both	FPG, HbA_{1c}	Lab	Lab
Chennai Urban Population Study (CUPS)	India	1996-1999	310	Community	Urban	FPG, 2hOGTT	Lab	-
The Hisayama Study	Japan	2002-2003	3,212	Community	Rural	FPG, HbA_{1c}	Lab	Lab
National Health and Nutrition Survey	Japan	2005	1,360	National	Both	FPG, HbA_{1c}	Lab	Lab
National Health and Nutrition Survey	Japan	2006	1,520	National	Both	FPG, HbA_{1c}	Lab	Lab
National Health and Nutrition Survey	Japan	2007	1,413	National	Both	FPG, HbA_{1c}	Lab	Lab
National Health and Nutrition Survey	Japan	2008	1,618	National	Both	FPG, HbA_{1c}	Lab	Lab
National Health and Nutrition Survey	Japan	2009	1,668	National	Both	FPG, HbA_{1c}	Lab	Lab
National Health and Nutrition Survey	Japan	2010	1,430	National	Both	FPG, HbA_{1c}	Lab	Lab
Observation des Risques et de la Sante Cardio-Vasculaire au Luxembourg (ORISCAV-LUX)	Luxembourg	2007-2009	1,200	National	Both	FPG, HbA_{1c}	Lab	Lab
Healthy Life in an Urban Setting (HELIUS)	Netherlands	2011-2013	1,380	Community	Urban	FPG, HbA_{1c}	Lab	Lab

Survey name	Country	Survey year(s)	Participants ≥ 18 years	Level of representative-ness	Rural, urban or both	Biomarkers available	Glucose measurement	HbA_{1c} measurement
Life Course Study in Cardiovascular Disease Epidemiology	Philippines	2009	623	Subnational	Both	FPG, 2hOGTT	Lab	-
Study of prevalence and control of CVD risk factors in the general adult population in Poland (NATPOL)	Poland	2011	535	National	Both	FPG, HbA_{1c}	Lab	Lab
Singapore Health Study 2012	Singapore	2012-2013	1,712	National	Both	FPG, HbA_{1c}	Lab	Lab
Taiwanese Survey on Hypertension, Hyperglycemia and Hyperlipidemia	Taiwan	2002	6,015	National	Both	FPG, HbA_{1c}	Lab	Lab
Taiwanese Survey on Hypertension, Hyperglycemia and Hyperlipidemia	Taiwan	2007	4,023	National	Both	FPG, HbA_{1c}	Lab	Lab
Edinburgh Artery Study	UK	1987-1988	342	Community	Urban	FPG, 2hOGTT	Lab	-

Webfigure 1: Regional random effects in the regression of diabetes prevalence defined based on HbA_{1c} ($HbA_{1c} \geq$ 6.5% or history of diabetes or using insulin or oral hypoglycaemic agents) against diabetes prevalence defined based on FPG (FPG $\geq$ 7.0 mmol/L or history of diabetes or using insulin or oral hypoglycaemic agents)

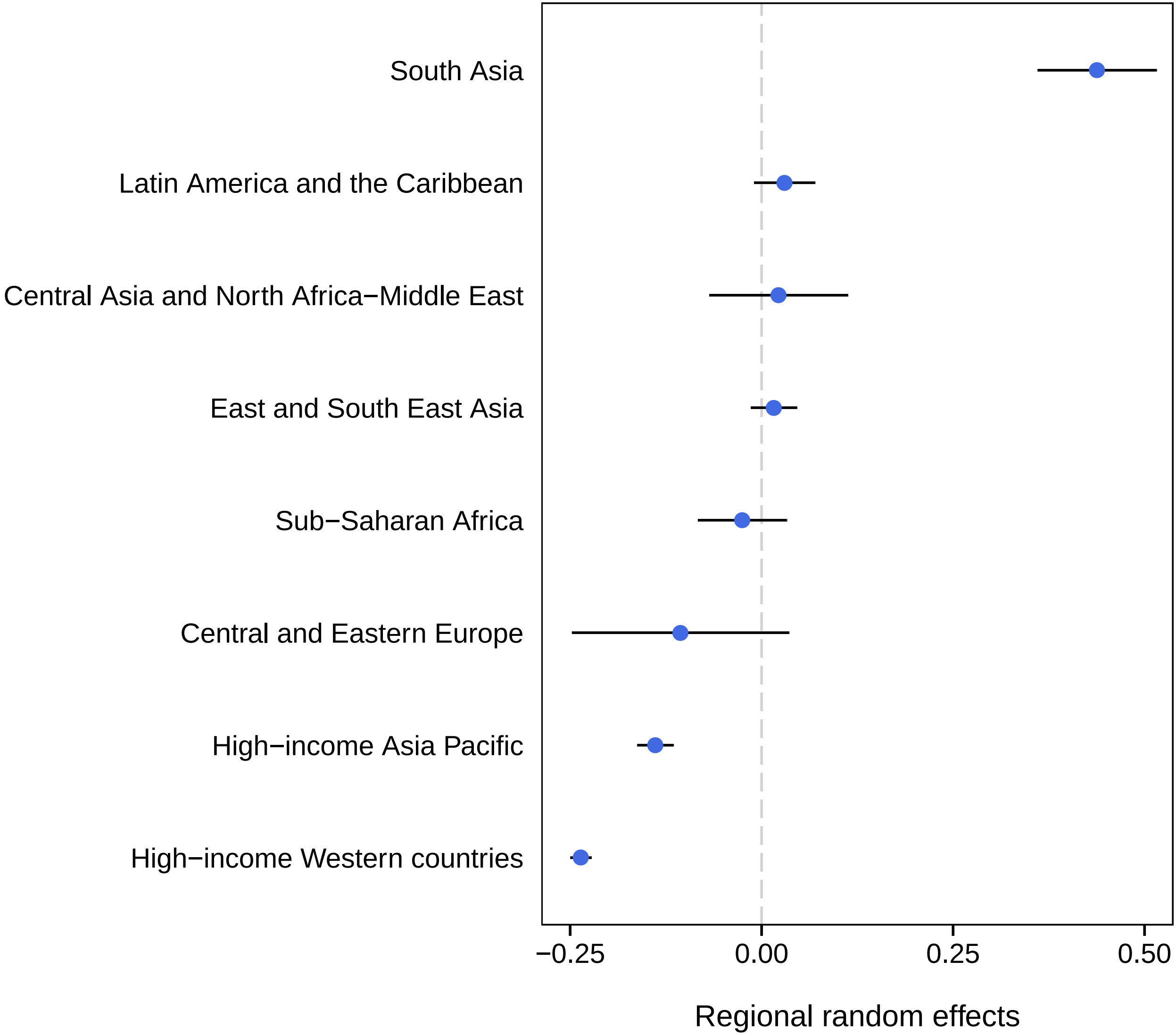

South Asia
Latin America and the Caribbean
Central Asia and North Africa−Middle East
East and South East Asia
Sub−Saharan Africa
Central and Eastern Europe
High−income Asia Pacific
High−income Western countries
−0.25
0.00
0.25
0.50
Regional random effects

Webfigure 2: Random effect meta-analysis of diabetes diagnostic sensitivity of HbA_{1c} ≥ 6.5% vs. FPG ≥ 7.0 mmol/L in previously-undiagnosed participants

study_name	country	study_years		Sensitivity (95% CI)	% Weight
Costa Rican Longevity and Healthy Aging Study Pre-1945 Cohort Wave 1	Costa Rica	2004-2006		12.99 (8.04, 17.95)	8.98
Harmonizing Equation of Risk in Mediterranean countries EXtremadura	Spain	2007-2009		23.66 (15.02, 32.29)	2.95
Study on Nutrition and Cardiovascular Risk in Spain	Spain	2008-2010		24.86 (18.57, 31.16)	5.56
Population Health Survey in Greenland	Greenland	2005-2010		25.00 (17.93, 32.07)	4.41
Bruneck Study	Italy	2000-2000		25.00 (7.68, 42.32)	0.73
The Australian Diabetes Obesity and Lifestyle Study 1999-2000	Australia	1999-2000		30.29 (24.49, 36.09)	6.55
Health Survey for England 2003	United Kingdom	2003-2003		40.00 (15.21, 64.79)	0.36
US NHANES 2007-2008	United States of America	2007-2008		40.68 (31.81, 49.54)	2.81
US NHANES 2011-2012	United States of America	2011-2012		44.55 (34.86, 54.25)	2.35
US NHANES 2001-2002	United States of America	2001-2002		44.57 (34.41, 54.72)	2.14
Metabolic Syndrome Study in Malaysia	Malaysia	2008-2008		47.96 (42.25, 53.67)	6.76
English Longitudinal Study of Ageing Wave 2 2004-2005	United Kingdom	2004-2005		50.00 (35.86, 64.14)	1.10
Cape Town Bellville South Cohort Study - Baseline evaluation I	South Africa	2008-2009		50.43 (41.30, 59.57)	2.64
US NHANES 2009-2010	United States of America	2009-2010		50.85 (41.83, 59.87)	2.71
US NHANES 2003-2004	United States of America	2003-2004		52.54 (39.80, 65.28)	1.36
China Health and Nutrition Study 2009	China	2009-2009		52.86 (48.08, 57.63)	9.67
US NHANES 2005-2006	United States of America	2005-2006		54.93 (43.36, 66.50)	1.65
MRC National Survey of Health and Development	United Kingdom	2006-2011		56.38 (46.36, 66.41)	2.19
US NHANES 1999-2000	United States of America	1999-2000		59.02 (46.67, 71.36)	1.45
Korea National Health and Nutrition Examination Survey	Republic of Korea	2011-2011		69.01 (61.41, 76.62)	3.81
Korea National Health and Nutrition Examination Survey	Republic of Korea	2012-2012		71.90 (64.77, 79.02)	4.34
CRONICAS Cohort Study	Peru	2009-2012		75.00 (65.74, 84.26)	2.57
Seychelles Heart Survey	Seychelles	2013-2014		76.92 (66.68, 87.17)	2.10
Baependi Heart Study	Brazil	2010-2013		77.78 (66.69, 88.87)	1.79
The Three city Study	France	2008-2010		78.26 (61.40, 95.12)	0.78
North West Adelaide Health Study	Australia	1999-2003		83.55 (77.66, 89.45)	6.35
Chennai Urban Rural Epidemiology Study	India	2001-2004		93.18 (88.88, 97.48)	11.92
Overall (I-squared = 97.6%, p = 0.000)				52.82 (51.33, 54.30)	100.00

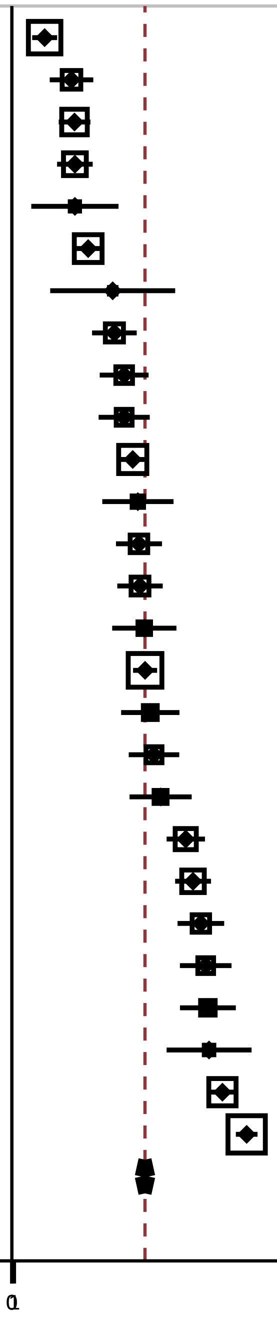

Webfigure 3: Random effect meta-analysis of diabetes diagnostic specificity of HbA_{1c} ≥ 6.5% vs. FPG ≥ 7.0 mmol/L in previously-undiagnosed participants

study_name	country	study_years	Specificity (95% CI)	% Weight
Seychelles Heart Survey	Seychelles	2013-2014	88.13 (86.16, 90.11)	0.03
The Three city Study	France	2008-2010	90.86 (88.56, 93.17)	0.02
Chennai Urban Rural Epidemiology Study	India	2001-2004	92.30 (91.15, 93.46)	0.09
Baependi Heart Study	Brazil	2010-2013	94.09 (92.85, 95.33)	0.08
CRONICAS Cohort Study	Peru	2009-2012	94.57 (93.74, 95.40)	0.17
Cape Town Bellville South Cohort Study - Baseline evaluation I	South Africa	2008-2009	94.57 (92.89, 96.25)	0.04
China Health and Nutrition Study 2009	China	2009-2009	96.74 (96.35, 97.13)	0.76
Metabolic Syndrome Study in Malaysia	Malaysia	2008-2008	96.98 (96.40, 97.56)	0.35
North West Adelaide Health Study	Australia	1999-2003	97.07 (96.53, 97.61)	0.41
Bruneck Study	Italy	2000-2000	97.23 (95.93, 98.53)	0.07
Korea National Health and Nutrition Examination Survey	Republic of Korea	2012-2012	97.62 (97.19, 98.04)	0.64
Korea National Health and Nutrition Examination Survey	Republic of Korea	2011-2011	97.97 (97.59, 98.35)	0.80
Population Health Survey in Greenland	Greenland	2005-2010	97.98 (97.46, 98.50)	0.44
MRC National Survey of Health and Development	United Kingdom	2006-2011	98.33 (97.72, 98.93)	0.32
US NHANES 2011-2012	United States of America	2011-2012	98.41 (97.86, 98.95)	0.39
Costa Rican Longevity and Healthy Aging Study Pre-1945 Cohort Wave 1	Costa Rica	2004-2006	98.44 (97.89, 98.99)	0.38
English Longitudinal Study of Ageing Wave 2 2004-2005	United Kingdom	2004-2005	98.91 (98.55, 99.26)	0.93
US NHANES 2007-2008	United States of America	2007-2008	98.92 (98.47, 99.37)	0.58
US NHANES 2009-2010	United States of America	2009-2010	99.05 (98.65, 99.44)	0.75
Health Survey for England 2003	United Kingdom	2003-2003	99.23 (98.62, 99.84)	0.32
US NHANES 2001-2002	United States of America	2001-2002	99.27 (98.90, 99.64)	0.87
US NHANES 2003-2004	United States of America	2003-2004	99.41 (99.07, 99.76)	0.99
US NHANES 2005-2006	United States of America	2005-2006	99.50 (99.17, 99.83)	1.10
US NHANES 1999-2000	United States of America	1999-2000	99.72 (99.48, 99.97)	1.96
Harmonizing Equation of Risk in Mediterranean countries EXtremadura	Spain	2007-2009	99.87 (99.73, 100.02)	5.86
Study on Nutrition and Cardiovascular Risk in Spain	Spain	2008-2010	99.91 (99.86, 99.97)	41.01
The Australian Diabetes Obesity and Lifestyle Study 1999-2000	Australia	1999-2000	99.92 (99.87, 99.98)	40.65
Overall (I-squared = 98.2%, p = 0.000)			99.74 (99.71, 99.78)	100.00

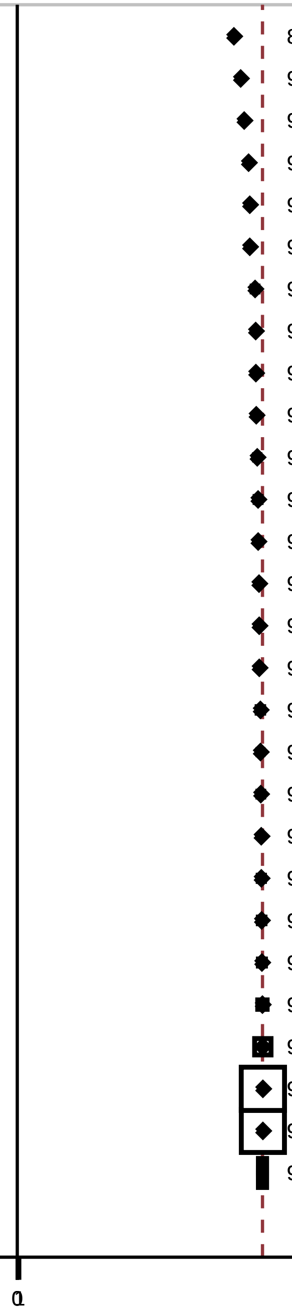

Webfigure 4: Random effect meta-analysis of diabetes diagnostic sensitivity of HbA_{1c} ≥ 6.5% vs. 2hOGTT ≥ 11.1 mmol/L in previously-undiagnosed participants

study_name	country	study_years	Sensitivity (95% CI)	% Weight
The Australian Diabetes Obesity and Lifestyle Study 1999-2000	Australia	1999-2000	20.73 (16.52, 24.93)	25.31
US NHANES 2007-2008	United States of America	2007-2008	22.76 (15.93, 29.58)	9.61
Population Health Survey in Greenland	Greenland	2005-2010	23.73 (16.05, 31.40)	7.59
US NHANES 2005-2006	United States of America	2005-2006	29.09 (20.60, 37.58)	6.21
US NHANES 2009-2010	United States of America	2009-2010	30.82 (23.33, 38.31)	7.98
US NHANES 2011-2012	United States of America	2011-2012	33.82 (25.87, 41.77)	7.08
Metabolic Syndrome Study in Malaysia	Malaysia	2008-2008	41.74 (36.62, 46.85)	17.10
Cape Town Bellville South Cohort Study - Baseline evaluation I	South Africa	2008-2009	56.32 (45.90, 66.74)	4.12
Chennai Urban Rural Epidemiology Study	India	2001-2004	78.70 (73.24, 84.16)	15.01
Overall (I-squared = 97.6%, p = 0.000)			37.16 (35.05, 39.28)	100.00

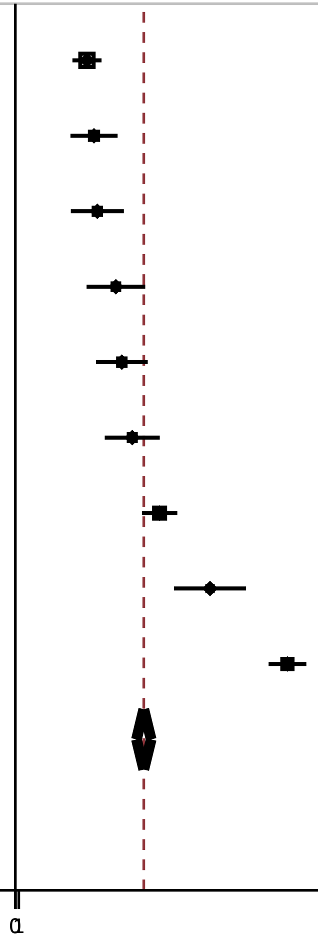

Webfigure 5: Random effect meta-analysis of diabetes diagnostic specificity of $HbA_{1c} \geq 6.5\%$ vs. 2hOGTT ≥ 11.1 mmol/L in previously-undiagnosed participants

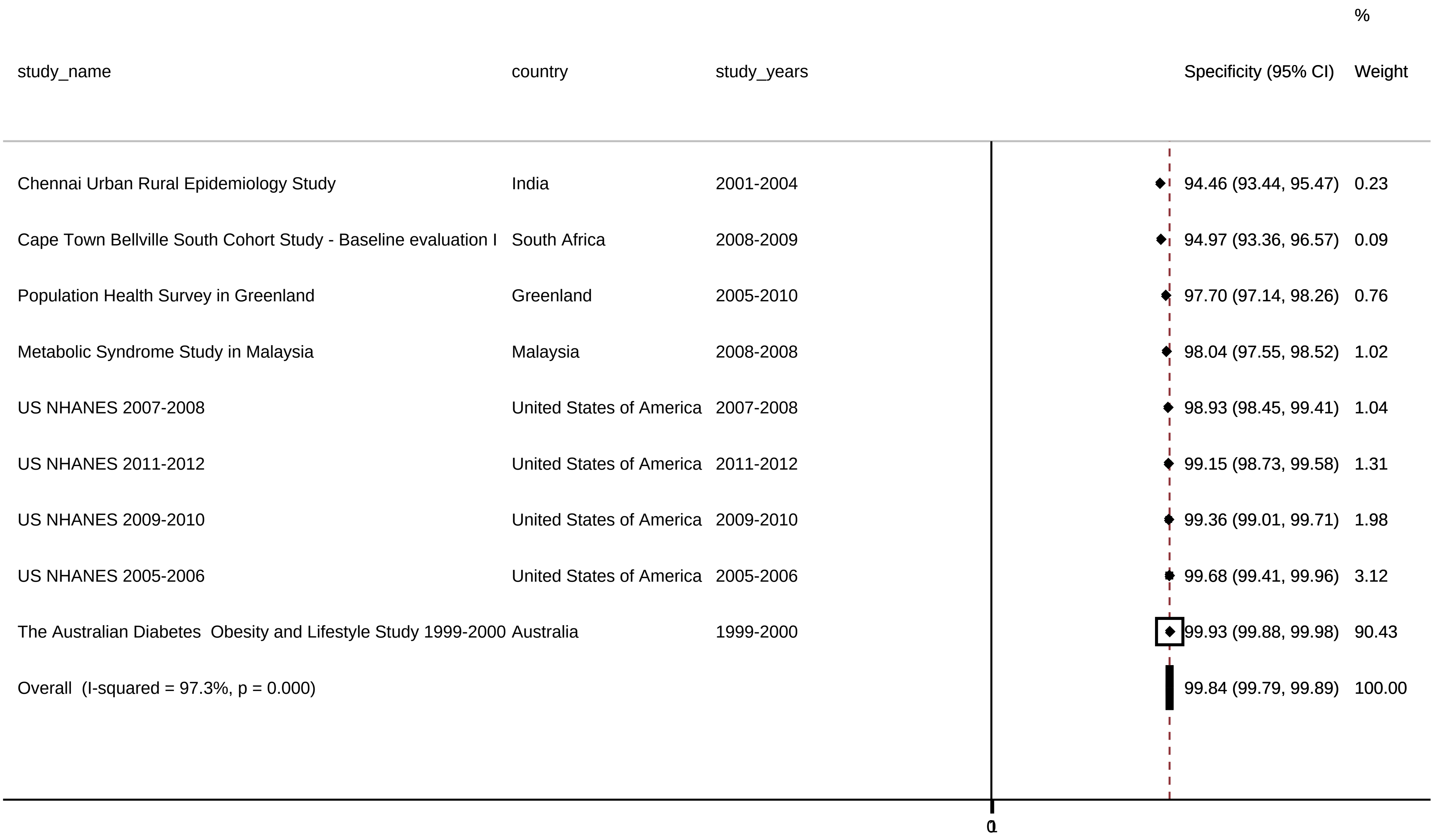
%
study_name
country
study_years
Specificity (95% CI)
Weight
Chennai Urban Rural Epidemiology Study
India
2001-2004
94.46 (93.44, 95.47)
0.23
Cape Town Bellville South Cohort Study - Baseline evaluation I
South Africa
2008-2009
94.97 (93.36, 96.57)
0.09
Population Health Survey in Greenland
Greenland
2005-2010
97.70 (97.14, 98.26)
0.76
Metabolic Syndrome Study in Malaysia
Malaysia
2008-2008
98.04 (97.55, 98.52)
1.02
US NHANES 2007-2008
United States of America
2007-2008
98.93 (98.45, 99.41)
1.04
US NHANES 2011-2012
United States of America
2011-2012
99.15 (98.73, 99.58)
1.31
US NHANES 2009-2010
United States of America
2009-2010
99.36 (99.01, 99.71)
1.98
US NHANES 2005-2006
United States of America
2005-2006
99.68 (99.41, 99.96)
3.12
The Australian Diabetes Obesity and Lifestyle Study 1999-2000
Australia
1999-2000
99.93 (99.88, 99.98)
90.43
Overall (I-squared = 97.3%, p = 0.000)
99.84 (99.79, 99.89)
100.00
0
1

Webfigure 6: Random effect meta-analysis of diabetes diagnostic sensitivity of HbA_{1c} ≥ 6.5% vs. FPG ≥ 7.0 mmol/L or 2hOGTT ≥ 11.1 mmol/L in previously-undiagnosed participants

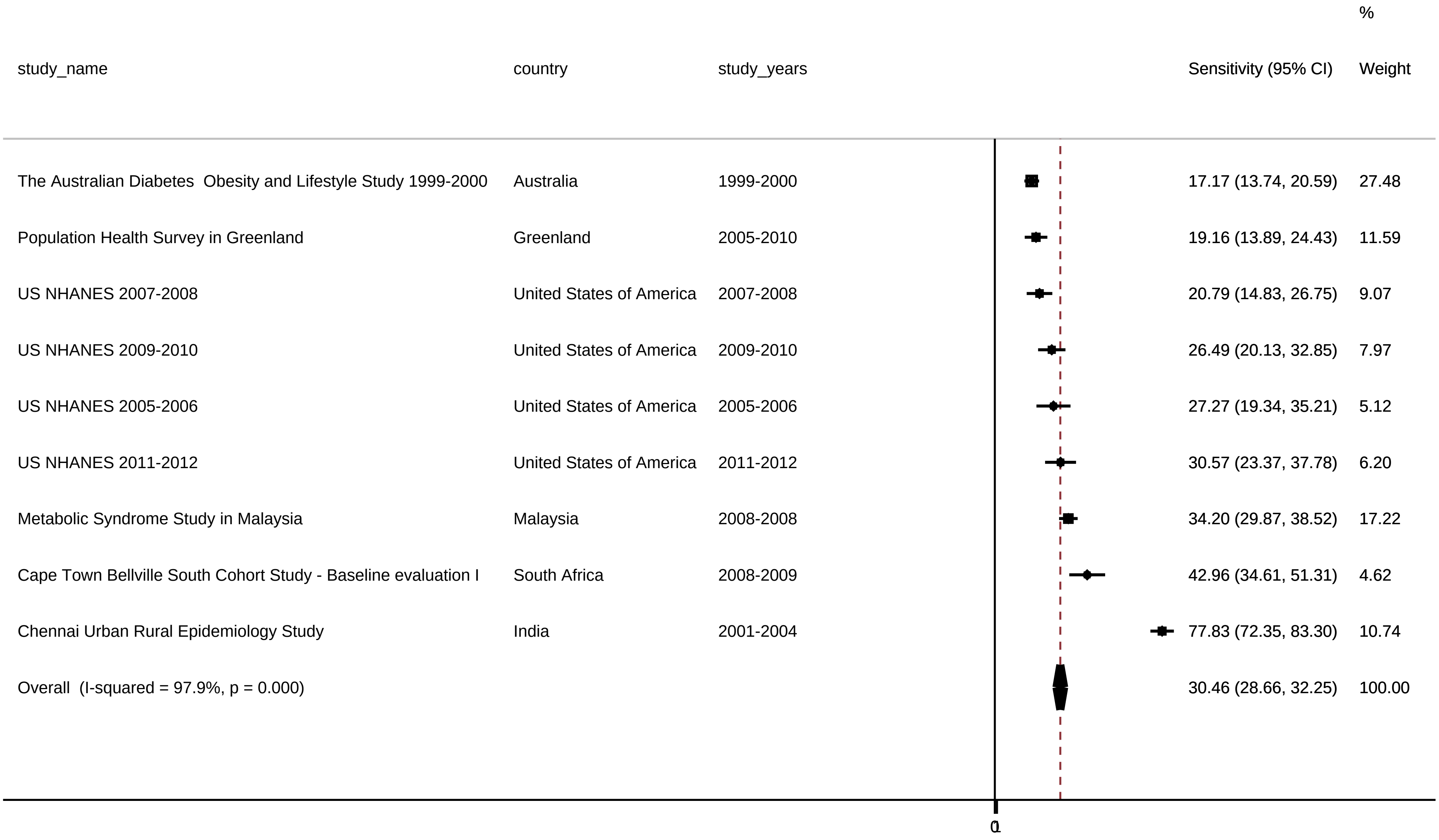

%
study_name
country
study_years
Sensitivity (95% CI)
Weight
The Australian Diabetes Obesity and Lifestyle Study 1999-2000 | Australia | 1999-2000 | 17.17 (13.74, 20.59) | 27.48
Population Health Survey in Greenland | Greenland | 2005-2010 | 19.16 (13.89, 24.43) | 11.59
US NHANES 2007-2008 | United States of America | 2007-2008 | 20.79 (14.83, 26.75) | 9.07
US NHANES 2009-2010 | United States of America | 2009-2010 | 26.49 (20.13, 32.85) | 7.97
US NHANES 2005-2006 | United States of America | 2005-2006 | 27.27 (19.34, 35.21) | 5.12
US NHANES 2011-2012 | United States of America | 2011-2012 | 30.57 (23.37, 37.78) | 6.20
Metabolic Syndrome Study in Malaysia | Malaysia | 2008-2008 | 34.20 (29.87, 38.52) | 17.22
Cape Town Bellville South Cohort Study - Baseline evaluation I | South Africa | 2008-2009 | 42.96 (34.61, 51.31) | 4.62
Chennai Urban Rural Epidemiology Study | India | 2001-2004 | 77.83 (72.35, 83.30) | 10.74
Overall (I-squared = 97.9%, p = 0.000) | 30.46 (28.66, 32.25) | 100.00
0
1

Webfigure 7: Random effect meta-analysis of diabetes diagnostic specificity of HbA_{1c} ≥ 6.5% vs. FPG ≥ 7.0 mmol/L or 2hOGTT ≥ 11.1 mmol/L in previously-undiagnosed participants

study_name	country	study_years	Specificity (95% CI)	% Weight
Chennai Urban Rural Epidemiology Study	India	2001-2004	92.22 (91.06, 93.38)	0.32
Cape Town Bellville South Cohort Study - Baseline evaluation I	South Africa	2008-2009	94.09 (92.39, 95.78)	0.15
Metabolic Syndrome Study in Malaysia	Malaysia	2008-2008	96.96 (96.38, 97.54)	1.28
Population Health Survey in Greenland	Greenland	2005-2010	97.58 (97.02, 98.15)	1.34
US NHANES 2011-2012	United States of America	2011-2012	98.48 (97.92, 99.04)	1.39
US NHANES 2007-2008	United States of America	2007-2008	98.71 (98.20, 99.22)	1.65
US NHANES 2009-2010	United States of America	2009-2010	99.01 (98.58, 99.43)	2.42
US NHANES 2005-2006	United States of America	2005-2006	99.69 (99.43, 99.96)	6.03
The Australian Diabetes Obesity and Lifestyle Study 1999-2000	Australia	1999-2000	99.86 (99.79, 99.94)	85.41
Overall (I-squared = 98.0%, p = 0.000)			99.69 (99.63, 99.76)	100.00

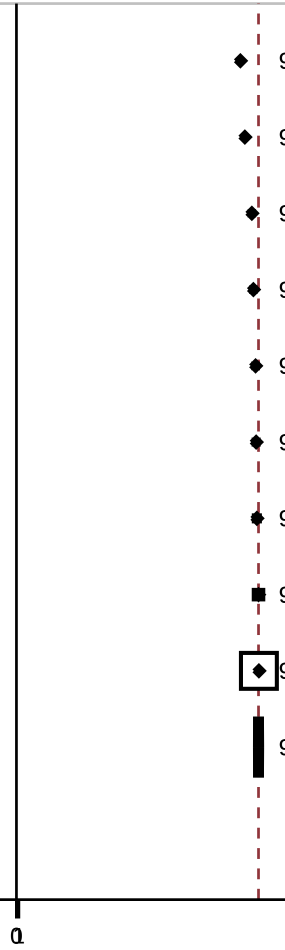

Webfigure 8: Random effect meta-analysis of diabetes diagnostic sensitivity of FPG ≥ 7.0 mmol/L vs. 2hOGTT ≥ 11.1 mmol/L in previously-undiagnosed participants

study_name	country	study_years	Sensitivity (95% CI)	% Weight
US NHANES III	United States of America	1988-1994	25.34 (22.20, 28.48)	13.53
Epidemiological survey of Kiribati	Kiribati	1981-1981	29.63 (12.41, 46.85)	0.45
Survey in Ramallah District Rural	Occupied Palestinian Territorry	1996-1998	30.77 (5.68, 55.86)	0.21
US NHANES II	United States of America	1976-1980	35.48 (28.61, 42.36)	2.82
The Australian Diabetes Obesity and Lifestyle Study 1999-2000	Australia	1999-2000	36.87 (31.87, 41.87)	5.33
Population Health Survey in Greenland	Greenland	2005-2010	37.29 (28.56, 46.01)	1.75
US NHANES 2009-2010	United States of America	2009-2010	37.41 (29.59, 45.24)	2.18
US NHANES 2007-2008	United States of America	2007-2008	39.31 (31.36, 47.26)	2.11
Bruneck Study	Italy	1990-1990	41.18 (24.63, 57.72)	0.49
Hong Kong Cardiovascular Risk Factor Prevalence Study 1995-1996	China (Hong Kong SAR)	1995-1996	43.04 (35.32, 50.76)	2.23
US NHANES 2011-2012	United States of America	2011-2012	44.85 (36.49, 53.21)	1.91
Metabolic Syndrome Study in Malaysia	Malaysia	2008-2008	44.97 (39.82, 50.13)	5.02
Survey in Ramallah District Urban	Occupied Palestinian Territorry	1996-1998	46.15 (19.05, 73.25)	0.18
CAMDI	Costa Rica	2004-2004	47.06 (23.33, 70.79)	0.24
New Delhi Birth Cohort	India	1999-2002	47.62 (26.26, 68.98)	0.29
CAMDI	Nicaragua	2003-2004	48.39 (35.95, 60.83)	0.86
US NHANES 2005-2006	United States of America	2005-2006	48.65 (39.35, 57.95)	1.54
Hertfordshire Cohort Study	United Kingdom	1999-2004	48.78 (42.53, 55.03)	3.41
CAMDI	Honduras	2003-2004	51.61 (34.02, 69.20)	0.43
Epidemiological survey of Kiribati	Kiribati	1981-1981	51.85 (42.43, 61.28)	1.50
CAMDI	Belize	2004-2005	54.79 (43.38, 66.21)	1.02
Mauritius non communicable disease survey	Mauritius	1987-1987	56.94 (51.78, 62.11)	4.99
Chennai Urban Rural Epidemiology Study	India	2001-2004	57.73 (51.20, 64.25)	3.13
The Funafuti Survey	Tuvalu	1976-1976	60.00 (35.21, 84.79)	0.22
CAMDI	Guatemala	2001-2002	62.07 (44.41, 79.73)	0.43
Mauritius non communicable diseases survey	Mauritius	2009-2009	63.37 (59.22, 67.53)	7.71
Mauritius non communicable disease survey	Mauritius	1998-1998	63.83 (59.83, 67.84)	8.31
Mauritius non communicable disease survey	Mauritius	1992-1992	64.54 (60.36, 68.73)	7.61
Cape Town Bellville South Cohort Study - Baseline evaluation I	South Africa	2008-2009	65.91 (56.01, 75.81)	1.36
High prevalence of diabetes and CVD risk factors associated with urbanization in India. Diabetes Care 2008	India	2006-2006	72.97 (68.27, 77.66)	6.05
Trends in the prevalence and incidence of non-insulin-dependent diabetes mellitus and impaired glucose tolerance	Nauru	1987-1987	79.49 (70.53, 88.45)	1.66
Trends in the prevalence and incidence of non-insulin-dependent diabetes mellitus and impaired glucose tolerance	Nauru	1982-1982	79.72 (73.13, 86.31)	3.07
Trends in the prevalence and incidence of non-insulin-dependent diabetes mellitus and impaired glucose tolerance	Nauru	1994-1994	91.57 (87.49, 95.65)	8.00
Overall (I-squared = 96.9%, p = 0.000)			54.42 (53.26, 55.57)	100.00

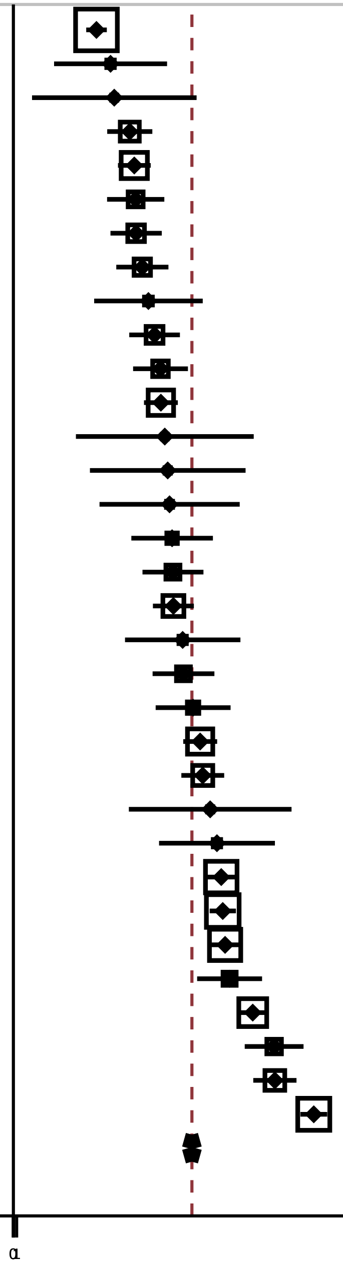

Webfigure 9: Random effect meta-analysis of diabetes diagnostic specificity of FPG ≥ 7.0 mmol/L vs. 2hOGTT ≥ 11.1 mmol/L in previously-undiagnosed participants

study_name	country	study_years	Specificity (95% CI)	% Weight
Trends in the prevalence and incidence of non-insulin-dependent diabetes mellitus and impaired glucose tolerance	Nauru	1994-1994	92.73 (91.10, 94.35)	0.20
Cape Town Bellville South Cohort Study - Baseline evaluation I	South Africa	2008-2009	93.30 (91.46, 95.13)	0.16
Trends in the prevalence and incidence of non-insulin-dependent diabetes mellitus and impaired glucose tolerance	Nauru	1987-1987	93.32 (91.59, 95.06)	0.18
Population Health Survey in Greenland	Greenland	2005-2010	96.51 (95.82, 97.20)	1.12
Hertfordshire Cohort Study	United Kingdom	1999-2004	96.66 (95.96, 97.36)	1.07
Metabolic Syndrome Study in Malaysia	Malaysia	2008-2008	96.66 (96.04, 97.29)	1.35
Mauritius non communicable diseases survey	Mauritius	2009-2009	96.92 (96.42, 97.41)	2.16
Trends in the prevalence and incidence of non-insulin-dependent diabetes mellitus and impaired glucose tolerance	Nauru	1982-1982	97.16 (96.15, 98.16)	0.52
New Delhi Birth Cohort	India	1999-2002	97.16 (96.29, 98.03)	0.70
Mauritius non communicable disease survey	Mauritius	1992-1992	97.47 (97.05, 97.89)	2.97
Mauritius non communicable disease survey	Mauritius	1998-1998	98.08 (97.66, 98.50)	2.99
US NHANES 2009-2010	United States of America	2009-2010	98.08 (97.48, 98.68)	1.48
Epidemiological survey of Kiribati	Kiribati	1981-1981	98.11 (97.44, 98.78)	1.17
US NHANES 2007-2008	United States of America	2007-2008	98.15 (97.52, 98.77)	1.34
Survey in Ramallah District Urban	Occupied Palestinian Territorry	1996-1998	98.44 (97.43, 99.45)	0.52
CAMDI	Belize	2004-2005	98.46 (97.77, 99.15)	1.12
Mauritius non communicable disease survey	Mauritius	1987-1987	98.56 (98.20, 98.92)	4.17
US NHANES 2011-2012	United States of America	2011-2012	98.82 (98.31, 99.32)	2.08
CAMDI	Costa Rica	2004-2004	98.83 (98.15, 99.52)	1.13
US NHANES III	United States of America	1988-1994	98.87 (98.57, 99.17)	5.97
The Australian Diabetes Obesity and Lifestyle Study 1999-2000	Australia	1999-2000	98.92 (98.72, 99.13)	13.09
CAMDI	Honduras	2003-2004	99.03 (98.46, 99.60)	1.62
US NHANES II	United States of America	1976-1980	99.12 (98.81, 99.43)	5.54
Bruneck Study	Italy	1990-1990	99.16 (98.54, 99.78)	1.37
The Funafuti Survey	Tuvalu	1976-1976	99.25 (98.40, 100.10)	0.74
US NHANES 2005-2006	United States of America	2005-2006	99.31 (98.90, 99.72)	3.17
CAMDI	Nicaragua	2003-2004	99.40 (99.00, 99.79)	3.41
Hong Kong Cardiovascular Risk Factor Prevalence Study 1995-1996	China (Hong Kong SAR)	1995-1996	99.40 (99.10, 99.70)	5.75
High prevalence of diabetes and CVD risk factors associated with urbanization in India. Diabetes Care 2008	India	2006-2006	99.42 (99.23, 99.61)	14.72
CAMDI	Guatemala	2001-2002	99.50 (99.01, 99.99)	2.19
Epidemiological survey of Kiribati	Kiribati	1981-1981	99.57 (99.16, 99.99)	3.05
Survey in Ramallah District Rural	Occupied Palestinian Territorry	1996-1998	99.64 (99.15, 100.14)	2.15
Chennai Urban Rural Epidemiology Study	India	2001-2004	99.75 (99.53, 99.97)	10.83
Overall (I-squared = 94.4%, p = 0.000)			98.90 (98.83, 98.97)	100.00

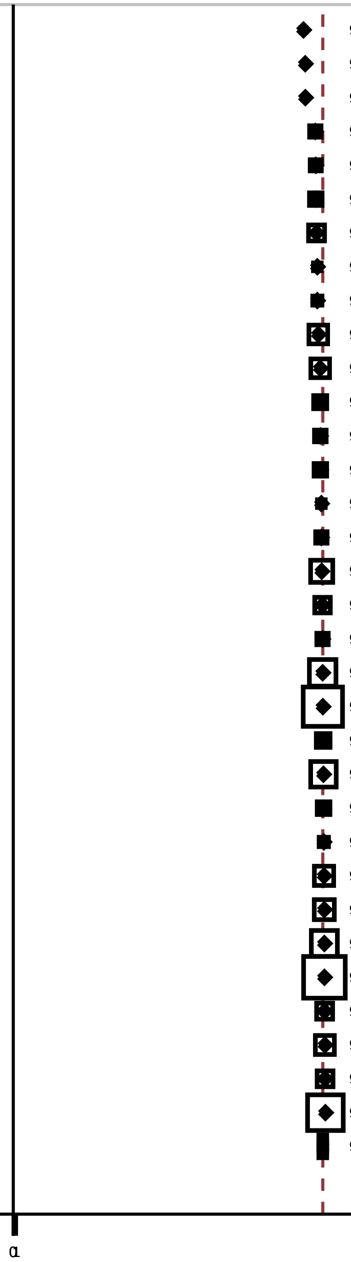

References

1. Sacks DB, Arnold M, Bakris GL, et al. Guidelines and recommendations for laboratory analysis in the diagnosis and management of diabetes mellitus. *Clin Chem* 2011; **57**(6): e1-e47.
